# JAK-STAT pathway-associated skin diseases: a refined functional framework for inflammatory skin diseases

**DOI:** 10.3389/fimmu.2026.1851255

**Published:** 2026-06-18

**Authors:** Bingrun Li, Xiaoting Song, Cunhao Shan, Junchen He, Zihan Wang, Katarina Stevanovic, Torsten Zuberbier, Zuotao Zhao, Litao Zhang

**Affiliations:** 1 Graduate School, Tianjin University of Traditional Chinese Medicine, Tianjin, China; 2 Department of Dermatology, Tianjin Academy of Traditional Chinese Medicine Affiliated Hospital, Tianjin, China; 3 Department of Dermatology, Beijing Chaoyang Hospital, Capital Medical University, Beijing, China; 4 Fraunhofer Institute for Translational Medicine and Pharmacology (ITMP), Allergology and Immunology, Berlin, Germany; 5 Institute of Allergology, Charité-Universitätsmedizin Berlin, corporate member of Freie Universität Berlin and Humboldt-Universität zu Berlin, Berlin, Germany

**Keywords:** autoimmune disease, cytokines, inflammatory skin diseases, JAK-STAT signaling pathway, JAK inhibitors, helper T cells

## Abstract

The Janus kinase (JAK)/signal transducer and activator of transcription (STAT) signaling pathway is involved in the pathogenesis of a variety of inflammatory and autoimmune skin diseases, such as atopic dermatitis (AD), psoriasis, alopecia areata (AA), vitiligo, etc. Many cytokines are involved in the occurrence and development of these diseases through the JAK-STAT-mediated intracellular signaling. Small molecule JAK inhibitors (JAKi) have demonstrated substantial efficacy in the treatment of many diseases, especially in inflammatory skin diseases. For example, abrocitinib and upadacitinib have been approved for the treatment of moderate-to-severe AD, and baricitinib has been approved for the treatment of severe AA. Meanwhile, clinical and preclinical studies of various JAKis for other skin diseases are ongoing. However, the traditional classification of inflammatory skin diseases according to the T helper (Th) immune axis fails to distinguish between cytokine signals that are mechanistically dependent on the JAK-STAT pathway and those that are not, thus limiting its utility for guiding targeted therapy. Accordingly, we propose a functional refinement framework that integrates JAK-dependent cytokine modules into the established Th immune axis system. This article reviews the pathogenesis of “JAK-STAT pathway-associated skin diseases” and the available efficacy evidence of JAKi therapy, aiming to improve the mechanistic understanding and rational clinical application of JAKi and provide valuable references for clinical diagnosis and treatment.

## 1 Introduction

Cytokines are soluble, low molecular weight signaling polypeptides, secreted by activated immune cells and some interstitial cells. These include interleukins (IL), interferons (IFN), tumor necrosis factor (TNF), colony-stimulating factor (CSF), chemokines, transforming growth factor β (TGF-β), etc. They play a role in cell growth and differentiation, immune response, wound healing, and tumor growth (1, 2).

The biological effects of cytokines are pleiotropic, overlapping, and interconnected. A single cytokine can act on a variety of target cells and conversely, different cytokines can also act on the same target cell, forming a highly complex signaling network. Cytokines exert their biological effects through receptor-mediated intracellular signaling pathways (3, 4).

Over the past two decades, biologics targeting specific cytokines or their receptors have shown significant success in autoimmune and inflammatory diseases. Notable examples for the treatment of psoriasis include TNF-α inhibitors, IL-12/23 inhibitors, IL-17A inhibitors, dupilumab (IL-4/IL-13 inhibitor), tralokinumab (IL-13 inhibitor), and nemoluzumab (IL-31 inhibitor) for the treatment of atopic dermatitis (AD) (5–7). Despite the remarkable efficacy of these biologics, treatments targeting single cytokines are not sufficient for diseases involving multiple cytokines. On the other hand, the potential immunogenicity of biologics raises concern about efficacy attenuation over extended treatment periods. Furthermore, the requirements from injectable drug delivery and the need for low temperature preservation pose significant logistical challenges for administration of biologics (8).

In the past 20 years, with the increasing understanding of intracellular pathways downstream of cytokine receptors have highlighted the therapeutic potential of inhibiting intracellular enzymes, such as receptor-associated kinases (9). Small molecule drug targeting receptor-associated kinases can modulate multiple cytokines simultaneously, potentially offering broader and more potent effect than biologics targeting a single cytokine (10, 11). This approach offers a promising new class of treatment options for inflammatory and autoimmune diseases involving complex cytokine interactions.

In traditional classification systems, dermatologic disorders were classified based on etiology into categories such as infectious inflammation, non-infectious inflammation, neoplastic disorders. They have also been categorized into type 1, 2, 3, and 4 inflammatory diseases based on the type of inflammation (12). However, with the better understanding of molecular mechanism underlying these diseases and the demonstrated efficacy of kinase-targeting small molecule drugs in various skin diseases, it has become a trend to classify these diseases according to molecular mechanism. Traditionally, the immunological classification of inflammatory skin diseases has relied primarily on Th1, Th2, and Th17 cell phenotypes; however, this framework remains inadequate in explaining disease heterogeneity, cross-disease commonalities, and differences in treatment responses: a single disease may exhibit mixed infiltration of multiple phenotypes (e.g., the coexistence of Th2 and Th17 cells in atopic dermatitis, and Th17/Th1 coexistence in psoriasis), while different diseases may also share similar cellular phenotypic characteristics (13). As a common downstream signaling axis for multiple Th cell factors, the JAK-STAT pathway—independent of upstream cellular phenotypes—directly regulates the final effector output of skin inflammation, clinical phenotypes, and treatment responses. Utilizing the JAK-STAT pathway as an independent analytical dimension facilitates a unified understanding of the mechanisms underlying inflammatory skin diseases and enables precision medicine, rendering it a valuable topic for review studies. Accordingly, we propose a functional refinement framework for “JAK-STAT pathway-associated skin diseases”, analyzing the associations between cytokines and the JAK-STAT pathway across different cellular phenotypes, to better characterize the mechanistic heterogeneity of inflammatory skin diseases and their potential therapeutic responsiveness to JAKis.

## 2 Discovery, composition, and function of the JAK-STAT pathway

**Figure 1:**
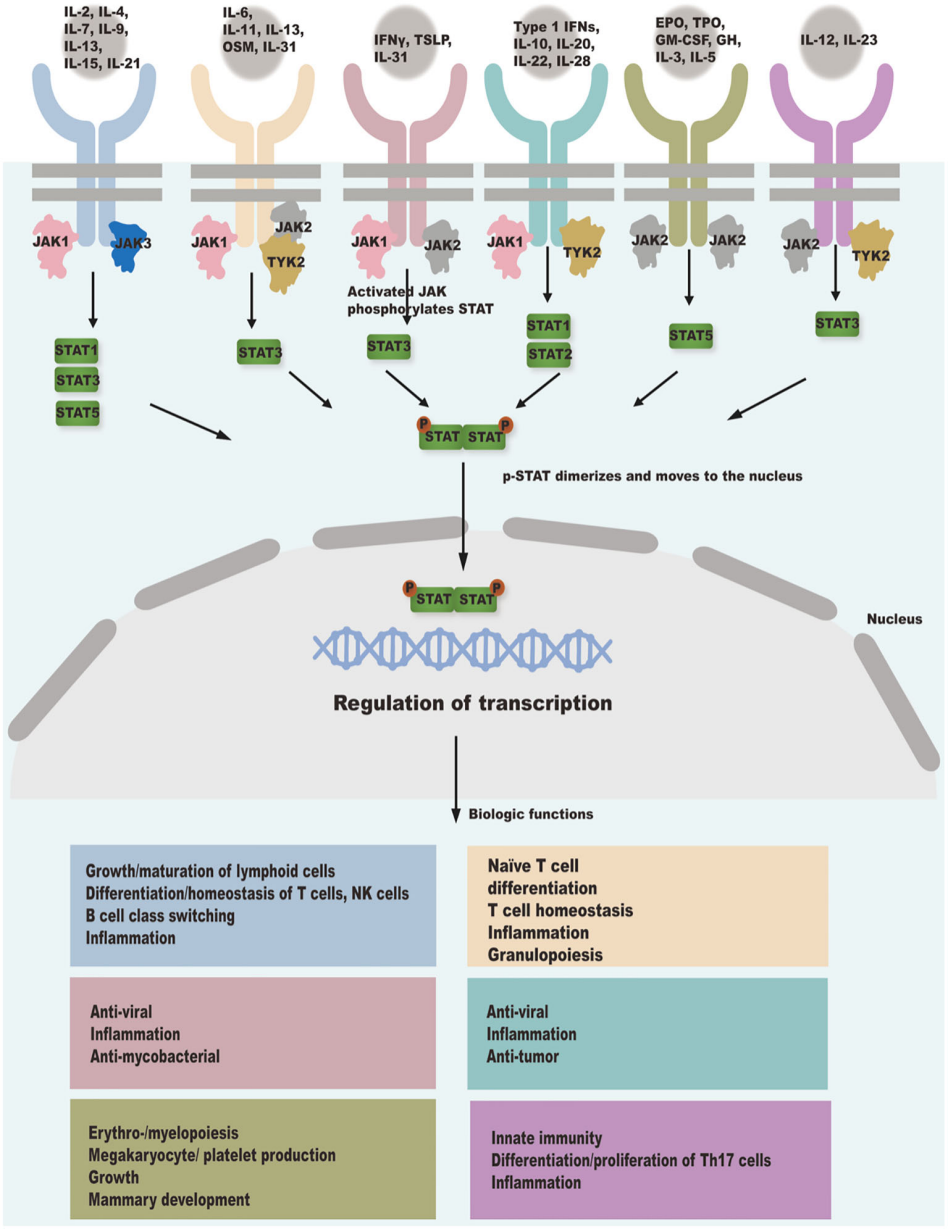
The JAK/STAT signaling pathway.

The JAK/STAT signaling pathway was first discovered when studying the downstream signaling of IFNs (14). A cytoplasmic tyrosine kinase induced by IFN-α further phosphorylate and activate the transcriptional activator interferon-stimulated gene factor 3 (ISGF3), mediating intracellular signaling of IFN-α (15). The JAK family belongs to a family of cytokine receptor-associated tyrosine kinases, and it has four distinct subtypes in mammals: JAK1, JAK2, JAK3, and tyrosine kinase 2 (TYK2) (16). Different JAKs mediate different cytokine signaling (**Table 1**) (17–19). JAK3 is predominantly expressed in hematopoietic cells and other three isoforms are ubiquitously expressed in tissues and cells throughout the body (20). STAT family members are activated by phosphorylation by JAKs, and then form homo- or heterodimers that translocate to the cell nucleus where they act as transcription activators. The STAT family of transcription factors comprises seven members: STAT1, STAT2, STAT3, STAT4, STAT5a, STAT5b, and STAT6 (21). In the JAK-STAT signaling pathway, cytokines or growth factors bind to their cognate receptors to form receptor-ligand complexes, resulting in conformational changes of receptors, activating JAK kinases and cross-phosphorylation of other JAK family members intracellularly (22). This forms an SH2 domain which recruiting and phosphorylating STATs. Subsequently, phosphorylated STAT dimers enter the nucleus, activating the transcription of genes involved in the inflammatory response (**Figure 1**) (18, 23).

**Table 1:** Features of different JAKs.

JAKs	Function	Cytokine family	Cytokine and growth factor
JAK1	Involved in a variety of type I and type II cytokine receptor signaling	Cytokine of the γc receptor subunit	IL-2, IL-4, IL-7, IL-9, IL-15, IL-21, IL-13, TSLP
		Gp130 receptor family	IL-6, IL-11, IL-31, IL-27, OSM, CNTF, LIF, CT-1, NNT-1, IFN-γ, IFN-α/β, IFN-λ
		IL-10 family cytokine receptor	IL-10, IL-19, IL-20, IL-22, IL-24, IL-26
JAK2	Involved in the signal transduction of a variety of hematopoietic functions and growth-related cytokines.	B chain cytokine	IL-3, IL-5, GM-CSF, TSLP
		Gp130 receptor family	IL-6, IL-11, IL-31, OSM, IL-13, IL-12, IL-23, IL-27, IFN-γ, CNTF, LIF, CT-1, NNT-1, leptin, prolactin, EPO, TPO, GH
TYK2	Together with JAK1 and JAK2, it participates in a variety of cytokine signal transduction and autoimmune function	Gp130 receptor family	IFN-α/β, IFN-λ, IL-6, IL-11, IL-12, IL-23, IL-27
		IL-10 family cytokine receptor	IL-10, IL-19, IL-20, IL-22, IL-24, IL-26, IL-12, IL-13, IL-23, IL-28, IL-29
JAK3	It plays an important role in the normal development and function of the immune system	Cytokine of the γc receptor subunit	IL-2, IL-4, IL-7, IL-9, IL-15, IL-21, IL-13

IL, Interleukin; TSLP, Thymic stromal lymphopoietin; OSM, oncostatin M; CNTF, ciliary neurotrophic factor; LIF, leukemia inhibitory factor receptor; CT−1, cardiotrophin; NNT-1, novel neurotrophic factor 1; GM-CSF, granulocyte-macrophage colony-stimulating factor; GH: Growth hormone; EPO, erythropoietin; TPO, thrombopoietin.

## 3 Association of JAK-STAT pathway, immune response type, and different clinical phenotypes

Th cells are CD4-positive (CD4+) T lymphocytes that play a key role in activating other immune cells, such as B cells and cytotoxic T cells, as well as regulating immune responses (24). CD4+ helper T cells further differentiate into different subpopulations characterized by cytokine secretion and effector function, including Th1, Th2, Th17/Th22, and Treg (25). Many inflammatory skin diseases, such as psoriasis, AD, and allergic contact dermatitis, are characterized by distinct CD4+ Th cell responses (26).

Importantly, these cytokine pathways exhibit varying degrees of dependence on JAK-STAT signaling (27). Such differences may, in turn, contribute to the heterogeneity of disease, the clinical phenotype, and differential therapeutic responses to JAKis. Notably, certain effector cytokines, such as IL-17A and IL-17F, primarily signal through JAK-independent pathways, whereas upstream cytokines including IL-23 and IL-22 rely on JAK-dependent signaling mechanisms (28). **Table 2** summarizes various inflammatory subtypes in terms of their immunological components, molecular signaling pathways, and the clinical phenotype for each of the immune response patterns.

**Table 2:** Immune response patterns and JAK-STAT pathway.

Pattern	Type 1	Type 2	Type 3		Type 4
Fate-specifying Cytokines	IL-12	IL-4	IL-6 IL-23 TGF-β	IL-6, TNF α	IL-2, TGF-β
	JAK2, TYK2	JAKI, JAK3	JAK 1, JAK2, TYK 2	JAK 1, JAK2, TYK 2	JAK1, JAK3
	STAT4	STAT6	STAT 3	STAT 3	STAT3, STAT5
Th subsets	Th1/ILC1	Th2/ILC2	Th17/ILC3	Th 22	Treg
Cytokines from specific T subsets	IFN-γ TNF-α	IL-4 IL-5-JAK2 IL-13-JAK1, JAK2/TYK2-STAT3, 6 IL-31-JAK1/2-STAT3, 5	IL-17A IL-17F*	IL-22-JAK1/TYK2-STAT1,3,5	IL-10, TGF-β
Clinical phenotype	Lichenoid	Eczematous	Psoriatic		Fibrogenic, granulomatous
Related disease	Lichen planus, lupus erythematosus	Atopic dermatitis, bullous pemphigoid, pemphigus vulgaris	Psoriasis, palmoplantar pustulosis,		Morphea, scleromyxedema, sarcoidosis

*IL-17 effector signaling is primarily mediated through JAK-independent pathways.

## 4 The role of the JAK-STAT pathway in skin diseases and its therapeutic application

The JAK-STAT pathway plays a key role in the pathogenesis of many skin diseases as described below in detail for each indication. Some indications have already received approval for treatment with JAKis, including AD, psoriasis, AA, and vitiligo. Some JAKi therapies are still in the clinical trial stage or have been discussed only in case reports, as summarized in **Table 3**. Other skin conditions in which JAKi have been tried, which is listed in **Supplementary Table 1**.

**Table 3:** The role of JAK-STAT pathway in skin diseases and its therapeutic application.

	Role of JAK-STAT pathway in pathogenesis	Application status of JAKis
Atopic Dermatitis	A variety of T cells such as Th1, Th2, Th17 and Th22 are abnormally activated, and a variety of JAK-STAT pathway cytokines (IL-4, IL-13, IL-31, IL-22, TSLP, IFN-γ, etc.) are involved in the pathogenesis of AD	**Approved drugs:** Baricitinib (oral) JAK1/2 Upadacitinib (oral) JAK1 Abrocitinib (oral) JAK1 Ruxolitinib (topical) JAK1/2 Delgocitinib (topical) JAK1/JAK2/JAK3/TYK2 Ivarmacitinib (SHR0302) (oral) JAK1 **Clinical trials:** SHR0302 (topical) JAK1 (NCT04717310))
Psoriasis/psoriatic arthritis	Th17 cells and the IL-23/IL-17 axis are the key to the pathogenesis of psoriasis, in which the key cytokines IL-23 and IL-22 are mediated by the JAK-STAT pathway	**Approved drugs:** Tofacitinib (PSA) Upadacitinib (PsA) Deucravacitinib (oral) (plaque psoriasis) **Clinical trials:** Ropsacitinib (PF-06826647) TYK2 Brepocitinib (PF-06700841) TYK2 Envudeucitinib (ESK-001) TYK2 Zasocitinib (TAK-279) TYK2 Nomelcitinib (D-2570) TYK2 Baricitinib Pefecitinib (ASP015K) pan-JAK Abrocitinib Solcitinib (GSK2586184) JAK Itacitinib (INCB039110) JAK
Alopecia areata	Upregulation of class I histocompatibility complex and activation of autoreactive NKG2D CD8+ T cells lead to upregulation of JAK-STAT-dependent inflammatory cytokines, primarily IL-15 and IFN-γ.	**Approved drugs:** Baricitinib Ritlecitinib (PF-06651600) JAK3/TEC **Clinical trials:** Upadacitinib Ruxolitinib Brepocitinib(PF-06700841) **Case report:** Tofacitinib Abrocitinib
Vitiligo	IFN-γ secreted by CD8+ T cells is an important part of the pathogenesis and progression of vitiligo, and IL-15 can promote the production of CD8+ effector memory T cells, which is associated with disease recurrence, and both IFN-γ and IL-15 are dependent on the JAK-STAT pathway	**Approved drugs:** Ruxolitinib (topical) **Clinical trials:** Upadacitinib(NCT04927975, NCT06118411) Baricitinib(NCT05950542) Tofacitinib (NCT05293119) Povorcitinib(NCT06113471, NCT06113445, NCT04818346) Cerudulatinib (topical) Syk/JAK Ritlecitinib JAK3/TEC Ifidancitinib JAK1/JAK3 **Case report:** Delgocitinib (topical)
Chronic hand eczema	The pathogenesis shares similarities with AD, involving dysregulation of multiple inflammatory cytokines, such as IL-4, IL-13, IL-31, IL-22, and TSLP, whose signaling is mediated by the JAK-STAT pathway.	**Approved drugs:** Delgocitinib (Topical) **Clinical trials:** Ruxolitinib (topical) Upadacitinib **Case report** Baricitinib Tofacitinib
Systemic Lupus erythematosus	The pathogenesis is complex, characterized by an imbalance in Th1, Th2, Th17, and Treg cell regulation, and elevated levels of type I and II IFNs dependent on the JAK-STAT pathway	**Clinical trials:** Baricitinib Solcitinib (GSK2586184) R333 Tofacitinib Upadacitinib Deucravacitinib (POETYK SLE-2 - IM011-247) **Case report:** Ruxolitinib
Dermatomyositis	Type I IFNs that rely on the JAK-STAT pathway play an important role in the pathogenesis of dermatomyositis	**Clinical trial:** Brepocitinib **Case report:** Tofacitinib Ruxolitinib Baricitinib Upadacitinib
Sarcoidosis	IFN-γ, which relies on the JAK-STAT pathway, is a key cytokine in granuloma formation	**Case report:** Ruxolitinib Upatacitinib **Clinical trial:** Tofacitinib
Allergic contact dermatitis (ACD)	T cell activation and the cytokines it produces play an important role in the pathogenesis of ACD, including IL-12, IL-23, and other pruritus and inflammatory cytokines that rely on the JAK-STAT pathway	**Case report:** Abrocitinib (oral) Tofacitinib Delgocitinib (topical) Upadacitinib
Autoimmune bullous diseases (AIBD)	A variety of JAK-STAT pathway-dependent cytokines (IL-4, IL-6, IL-8, IL-10, IL-12, IL-15) are involved in the pathogenesis	**Case report:** Tofacitinib Upadacitinib
Chronic urticaria (CU)	A variety of JAK-STAT-related cytokines play a role in the pathogenesis of CU, such as IL-31, IL-4, IL-10, IL-9, IFN-γ, etc	**Clinical trial:** Tofacitinib **Case report:** Ruxolitinib Abrocitinib
Lichen planopilaris (LP)	The IFN-γ/CXCL10 axis is pivotal in the pathogenesis and chronic inflammation of LP. IFN-γ signaling via the JAK-STAT pathway (primarily involving JAK1 and JAK2) activates STAT1, driving the production of inflammatory mediators like CXCL10 and contributing to disease development.	**Clinical trial:** Tofacitinib Baricitinib Ruxolitinib (topical) **Case report:** Abrocitinib Ritlecitinib Upadacitinib Deucravacitinib
Livedoid vasculopathy (LV)	It is mainly associated with hypercoagulable state of blood and autoimmunity	**Case report:** Tofacitinib Baricitinib Upadacitinib Abrocitinib
Urticarial vasculitis (UV)	Immune complex-mediated type III hypersensitivity reactions. It shares the same clinical features as a variety of autoimmune diseases, and significantly elevated levels of IL-6 dependent on the JAK-STAT pathway have been observed in urticarial vasculitis	**Case report:** Tofacitinib Baricitinib

### 4.1 Atopic dermatitis

AD is a chronic inflammatory disease with severe itching. Various factors, including genetic susceptibility, skin barrier dysfunction, skin microbiota dysbiosis, and immune dysregulation, are involved in its pathogenesis (29). From normal-looking skin to acute and chronic phases, Th1, Th2, Th17 and Th22 cells are activated in varying degrees, releasing various cytokines that are mediated by the JAK-STAT signaling pathway as depicted in **Table 2** (30).

This transformation resulting in spongiform edema, epidermal hyperplasia (IL-22), inhibition of keratinocyte differentiation (IL-4, IL-13, IL-31, IL-22, IL-25), inducing keratinocytes to produce thymic stromal lymphoietin (TSLP), inhibiting the release of antimicrobial peptides (IL-4, IL-13), causing pruritus (IL-31, TSLP), chronic lichenification (IL-22, IFN-γ) and other heterogeneous pathological changes. Among the aforementioned cytokines, IL-4 and IL-13 signal primarily through JAK1-dependent pathways, whereas IL-31 is more closely associated with JAK1/2-mediated signaling (8, 31). Consequently, the JAK-STAT pathway has become an important target for the development of AD therapeutic drugs. A variety of oral and topical JAKi have been approved for the treatment of AD, and their latest trial results are outlined below. In clinical trials, the most commonly used clinical indicators for AD include Investigator’s Global Assessment (IGA) 0/1, Eczema Area and Severity Index (EASI)-50, EASI-75, EASI-90, Patient-Reported Numerical Rating Scale (PP-NRS)4, SCORing Atopic Dermatitis (SCORAD) (29).

#### 4.1.1 Oral JAKis

##### 4.1.1.1 Baricitinib

Baricitinib is a non-selective JAKi that was first approved in Europe for the treatment of moderate-to-severe AD in October 2020, and in general, the first JAKi approved for the treatment of AD in the world (32). Baricitinib monotherapy demonstrated superior efficacy compared to placebo after 16 weeks of treatment. Baricitinib 4 mg QD led to a significantly greater proportion of patients achieving IGA 0/1 (13.8%-31%) and EASI-75 (21.1%-48%), compared to a dose of baricitinib 2 mg QD (vIGA-AD 0/1, 10.6%-24%; EASI-75, 17.9%-29.5%) (33, 34). In combination with topical corticosteroids (TCS), 31% and 24% patients achieved IGA 0/1 at Week 16, in the 4mg and 2mg groups, respectively, compared to 15% in the placebo group (35). Meta-analyses further corroborate that baricitinib, administered as monotherapy or in combination with topical corticosteroids, demonstrated significant and consistent superiority over placebo across multiple doses in improving a spectrum of clinical outcomes in patients with AD (36). *Post-hoc* analyses of several phase 3 clinical trials revealed that baricitinib led to rapid and significant improvements in pruritus, itch impact on sleep, and patient-reported skin pain severity as early as 1 to 2 days after treatment initiation (37, 38). These effects may be related to the inhibition of JAK1/2-mediated cytokine signaling pathways involved in pruritus and neuroimmune regulation, including IL-31-associated signaling. Furthermore, baricitinib demonstrates sustained efficacy and a favorable safety profile in both adult and pediatric patients, maintaining improvements in disease control and patient-reported outcomes for up to 200 weeks in adults and 3.6 years in children, with no emergent safety signals observed during extended treatment (39–41).

##### 4.1.1.2 Upadacitinib

Upadacitinib is a selective JAK1 inhibitor. In both cell-free isolate enzyme activity assays and engineered cellular assay, it demonstrated greater selectivity for JAK1 over JAK2, JAK3 and TYK2 (42, 43). In January 2022, the FDA approved upadacitinib for the treatment of moderate-to-severe AD (42). In a phase III clinical trial of upadacitinib monotherapy, upadacitinib 15 mg QD and 30 mg QD significantly relieved pruritus after 1 week, and skin lesions after 2 weeks (44, 45). The rapid improvement in pruritus may be associated with the inhibition of JAK1-mediated cytokine pathways involved in itch signaling, particularly IL-4, IL-13, and IL-31-related signaling. The EASI-75 response rate at 16 weeks was 60.1% - 69.6% and 72.9% - 79.7% in 15 mg group and 30mg group, respectively (44); and each treatment successfully sustained efficacy responses through 52 weeks (46, 47). In another head-to-head clinical trial with dupilumab, the EASI-75 response rate was 43.7% at Week 2 using upadacitinib 30 mg QD, which was significantly higher than that in the dupilumab group (17.5%, p<0.001) (48). Recent findings from the Level Up study further demonstrated that upadacitinib achieved a significantly higher proportion of patients simultaneously reaching the composite endpoint of EASI 90 at Week 16 compared with dupilumab (19.9% vs. 8.9%, P<0.001) (49). Moreover, patients with inadequate response to dupilumab showed clinically meaningful improvement after switching to upadacitinib (50). When combined with TCS, at Week 16, the upadacitinib 30 mg plus TCS group (EASI-75, 77%; IGA 0/1, 59%) and upadacitinib 15 mg plus TCS group (EASI-75, 65%; IGA 0/1, 40%) achieved better clinical responses in comparison to the placebo group (EASI-75, 26%; IGA 0/1, 11%) (51).

##### 4.1.1.3 Abrocitinib

Abrocitinib, another highly selective JAK1 inhibitor, was approved by FDA in 2022 for the treatment of moderate-to-severe AD. Biochemical assays demonstrated substantially greater selectivity for JAK1 compared with JAK2, JAK3, and TYK2 (52). In phase III clinical trials, abrocitinib monotherapy 100mg QD and 200mg QD significantly relieved pruritus within 2 to 4 days, and achieved an EASI-75 response rate at 12 weeks of 40% - 44.5% and 61.0% - 63%, respectively (53–55). The rapid antipruritic effect may be associated with inhibition of JAK1-mediated cytokine pathways involved in itch signaling, including IL-4, IL-13, and IL-31-related pathways. Meta-analyses further confirmed the pronounced efficacy of abrocitinib in treating moderate-to-severe AD (56). During long-term treatment with abrocitinib (JADE EXEXT), clinically meaningful improvements in AD symptoms were observed, with the 200 mg regimen appearing more effective than the 100 mg regimen (IGA 0/1: 52% vs. 39%; EASI-75: 82% vs. 67%) (57, 58). In maintenance therapy in the responders (JADE REGIMEN), abrocitinib monotherapy significantly decreased flares, cumulative probability of experiencing a flare was 18.9% (95% CI, 14.2-24.9), 42.6% (95% CI, 36.3-49.5),and 80.9% (95% CI, 75.8-85.6) in the abrocitinib 200 mg, abrocitinib 100 mg, and placebo, respectively (59). Another phase III clinical trial, with the combination of TCS, an IGA response at Week 12 was observed in 48.4%, 36.6%, 36.5%, and 14.0% of patients in the 200 mg abrocitinib, 100 mg abrocitinib, dupilumab group, and placebo group, respectively. An EASI-75 response at Week 12 was observed in 70.3%, 58.7%, 58.1%, and 27.1%, respectively to the above groups (p<0.001 for both abrocitinib doses vs. placebo) (60). In a head-to-head clinical trial (JADE DARE) with dupilumab, the response rate of PP-NRS4 at Week 2 was 48.2%, and EASI-90 at Week 4 was 28.5% in the abrocitinib 200 mg group, which demonstrated numerically greater early clinical responses compared with dupilumab (25.5% and 14.6%, both p <0.0001) (61). Among prior dupilumab nonresponders, EASI-75 response rates at Week 12 after transitioning to abrocitinib were 80.0% and 67.7% for the 200 mg and 100 mg doses, respectively (62). In a *post hoc* analysis of JADE COMPARE and DARE, compared with dupilumab, a greater proportion of patients receiving abrocitinib 200 mg achieved T2T goals across assessed time points (63).

##### 4.1.1.4 Ivarmacitinib (SHR0302)

Ivarmacitinib is a selective JAK1 inhibitor approved in China for use in adult patients with moderate to severe AD (64). In a phase III clinical trial, patients with moderate-to-severe AD treated with ivarmacitinib 4 mg or 8 mg QD demonstrated significant efficacy, with symptom improvement observed as early as day 3, achieving IGA 0/1 response rates of 36.3% and 42.0%, respectively, and EASI-75 response rates of 54.0% and 66.1% at Week 16, compared to placebo (9.0% for IGA 0/1 and 21.6% for EASI-75; P<0.001) (65, 66). The rapid clinical improvement may reflect effective suppression of JAK1-dependent cytokine signaling pathways involved in type 2 inflammation and pruritus.

##### 4.1.1.5 Other JAKis

The following JAKis are currently in earlier clinical trial phases. Tofacitinib has a strong inhibitory effect on JAK1 and JAK3, and a weak inhibitory effect on JAK2 and TYK2 (67). There is no large-scale randomized controlled trial of tofacitinib for AD. In an open-label clinical trial, 6 patients with moderate-to-severe AD, who failed to respond to standard therapy, were treated with oral tofacitinib (5 patients with 5mg BID, 1 patient with 5 mg QD), and the SCORAD score decreased by 66.6% after 8–29 weeks of treatment (68). Further studies are required in order to clarify both the efficacy and the safety of the substance, taking into consideration its broader JAK inhibitory profile.

Gusacitinib (ASN002) is a dual inhibitor of SYK and JAK kinases. In a phase Ib trial, gusacitinib 20 mg, 40 mg, and 80 mg showed favorable tolerability and efficacy in the treatment of moderate-to- severe AD. The 80 mg group demonstrated significant pruritis relief after 8 days of treatment. The EASI-50 response rates of the gusacitinib 40 mg and 80 mg groups were 100% and 83%, respectively, which were significantly higher than that in the placebo group (22%). However, there was no significant improvement in EASI-75 (69). This finding suggests that dual SYK/JAK inhibition may exert differential effects on pruritus and inflammatory skin manifestations.

#### 4.1.2 Topical JAKis

##### 4.1.2.1 Delgocitinib

Delgocitinib 0.5% ointment is the first topical JAKi to ever be approved for the treatment of mild-to-moderate AD in January 2020 in Japan (70, 71). It is a non-selective JAKi, acting on JAK1, JAK2, JAK3 and TYK2 (70). In a phase III clinical trial, it decreased the modified EASI score by 44.3% at Week 4 in patients over 16 years of age with moderate-to-severe AD, which was significantly better than that in the vehicle group (increased by 1.7%, p<0.001). The skin lesions were improved and maintained during the continued treatment of 24 weeks (72). In a recent open-label, multicenter clinical study, 0.5% delgocitinib ointment demonstrated significant efficacy in patients with facial and neck AD inadequately controlled by topical corticosteroids or tacrolimus ointment. The incidence of adverse drug reactions, such as skin irritation and telangiectasia, was significantly reduced. Furthermore, results from the Patient Preference Questionnaire (PPQ) indicated that approximately 80% of patients preferred delgocitinib treatment (73).

##### 4.1.2.2 Ruxolitinib

In September 2021, the FDA approved ruxolitinib 1.5% cream for the treatment of mild-to-moderate AD. In two phase III clinical trial, 1.5% ruxolitinib cream BID demonstrated rapid antipruritic effects, achieving clinically meaningful itch reduction within 36 hours of application, and the EASI-75 response rates reached 61.8% - 62.1% at Week 8, which was significantly superior to that in the vehicle group (15.4% - 16.3%) (71, 72). Long-term studies confirmed that the therapy maintained disease control and quality of life improvements during a 52-week as-needed treatment period (73). In a recently completed phase III clinical trial in children aged 2–11 years, 1.5% ruxolitinib cream also exhibited significant efficacy; at Week 8, the IGA-TS rate was 56.5% (vs 10.8% for vehicle, p<0.0001) and the EASI-75 response rate reached 67.2% (vs 15.4% for vehicle, p<0.0001), with a favorable safety profile (74).

For difficult-to-manage head and neck lesions, ruxolitinib cream demonstrated rapid and significant improvement. At Week 8, among patients with baseline head and neck involvement, the head and neck EASI-75 response rate was 74.0% in the 1.5% ruxolitinib group, which was significantly superior to the vehicle group (31.6%, p<0.0001) (72). A decentralized phase 2 clinical trial further confirmed its efficacy and safety in facial and neck lesions (75). Furthermore, patient-reported outcome data indicated that, in addition to itch, skin pain was significantly reduced within 12 hours of the first application (p<0.03), and sleep disturbance scores were observed to improve significantly by Week 2 of treatment (73).

##### 4.1.2.3 Tofacitinib

In a phase II clinical study, the EASI score of adult AD patients with an affected area of 2% - 20% decreased by 87.1% after 4 weeks treatment with tofacitinib 2% ointment BID, which was significantly better than that in the vehicle group (20.9%, p<0.001) (74). In a recent phase III clinical study involving patients with mild-to-moderate AD, treatment with either 2% tofacitinib ointment or 1% pimecrolimus cream BID for 4 weeks resulted in significant reductions in EASI scores (9.94 - 3.32 and 8.76 - 3.04, respectively). Compared to the pimecrolimus group, the tofacitinib group demonstrated superior outcomes in the number of patients achieving EASI-75 at Weeks 2 and 4, EASI-90 at Week 4, and improvement in vIGA-AD score at Week 4 (75).

Collectively, these clinical findings further support the role of JAK-dependent cytokine signaling in pruritus, neuroimmune dysregulation, and site-specific inflammatory manifestations in AD. Furthermore, the utilization of topical JAKis may emerge as a valuable long-term therapeutic option for challenging-to-treat lesions in sensitive anatomical regions, such as the face and neck, where the application of prolonged topical corticosteroids is frequently constrained.

### 4.2 Psoriasis

Psoriasis is a chronic, immune-mediated disorder, in which chronic plaque psoriasis is the most common variant of psoriasis vulgaris, and psoriatic arthritis (PsA) is the major associated systemic manifestation (76). The pathogenesis of psoriasis is multifactorial, involving genetic predisposition, environmental triggers, and abnormal immune responses (5, 77). This complex interplay leads to abnormal proliferation of keratinocytes and an inflammatory response, affecting synovial cells and chondrocytes in the joints. About 20% - 30% of patients with psoriasis have comorbid arthritis or may develop PsA in the future (78).

A key pathogenic feature of psoriasis is the abnormal activation and infiltration of T lymphocytes in the epidermis and dermis. Damaged keratinocytes release antimicrobial peptides (AMPs) that activate plasmacytoid dendritic cells (pDCs), which further produce type I interferons and enable myeloid dendritic cells (mDCs) to activate the maturation and differentiation of Th1 and Th17 cells, which secrete various inflammatory cytokines and create a self-perpetuating inflammatory cycle. Importantly, not all psoriasis-associated cytokines are equally dependent on JAK-STAT signaling. While the key effector cytokines IL-17A and IL-17F primarily mediate downstream effector responses through JAK-independent pathways, upstream regulatory cytokines including IL-23 and IL-22 rely on JAK2/TYK2- and JAK1/TYK2-mediated signaling, respectively. Accordingly, type I interferons and IL-12 also signal through JAK-STAT pathway (79, 80).

At present, tofacitinib and upadacitinib have been approved for the treatment of PsA, while deucravacitinib has been approved for plaque psoriasis (81). Several other JAKis are in clinical or preclinical stages of research for psoriasis treatment (82). Since key downstream effector cytokines such as IL-17A and IL-17F primarily signal through JAK-independent pathways, the therapeutic efficacy of JAKis in psoriasis is mainly attributed to the inhibition of upstream cytokine modules involved in the pathogenesis of psoriasis—particularly the IL-23/TYK2 and IL-22 signaling pathways—rather than direct blockade of terminal effector responses (28). For psoriasis, the most commonly used clinical evaluations in clinical trials include Psoriasis Area and Severity Index (PASI)-50, PASI-75, PASI-90 and Physician Global Assessment (PGA).

#### 4.2.1 TYK2 inhibitors

##### 4.2.1.1 Deucravacitinib (BMS-986165)

Deucravacitinib is an allosteric TYK2 inhibitor and is currently the only JAKi approved by EMA and FDA for the treatment of plaque psoriasis (83). In a phase III study (POETYK PSO-1), the PASI-75 response rate was 58.4% after 16 weeks of treatment with deucravacitinib 6mg QD, which is significantly better than that of the placebo (12.7%, p<0.05) or the treatment with apremilast (35.1%) (84). In another phase III trial (POETYK PSO-2), the PASI-75 response rate of deucravacitinib 6mg QD for 16 weeks was 53.0%, which was significantly better than apremilast 30mg BID (9.4%, p<0.05) (85). In a phase IIIb/IIII trial for scalp psoriasis (NCT05478499), patients treated with deucravacitinib 6 mg QD for 16 weeks showed significantly better outcomes across scalp-specific efficacy measures compared to placebo. Specifically, the proportions achieving a scalp-specific PGA score of 0/1 and a Psoriasis Scalp Severity Index 90 response were 48.5% and 38.8%, respectively, significantly higher than 13.7% and 2.0% in the placebo group (p<0.0001). The mean improvement from baseline in the scalp-specific numeric rating scale for itch was -3.2, also significantly greater than the -0.7 improvement in the placebo group(p<0.0001). In the subgroup with a baseline static PGA (sPGA) score ≥3, 51.0% of patients in the deucravacitinib group achieved an sPGA 0/1 response, which remained significantly higher than the 4.3% observed in the placebo group (86).

##### 4.2.1.2 Ropsacitinib (PF-06826647)

Ropsacitinib is a selective TYK2 inhibitor. In a phase IIb clinical study, the PASI-90 response rates at Week 16 for patients with moderate-to-severe plaque psoriasis were 33.0% and 46.5% in the ropsacitinib 200mg group and the 400mg group, respectively (p<0.05) (87).

##### 4.2.1.3 Brepocitinib (PF-06700841)

Brepocitinib is a dual inhibitor of JAK1 and TYK2. In a phase IIa clinical study, patients with moderate -to-severe plaque psoriasis were treated with brepocitinib 30mg QD for 4 weeks as induction therapy, followed by maintenance therapy for 8 weeks. The response rate was 86.2%, which was significantly better than placebo (13.0%, p<0.05) (88).

##### 4.2.1.4 Envudeucitinib (ESK-001)

Envudeucitinib is a highly selective allosteric TYK2 inhibitor. In a phase II dose-ranging clinical study (STRIDE), the PASI-75 response rate at Week 12 for patients with moderate-to-severe plaque psoriasis was 64% in the envudeucitinib 40 mg BID group, which was significantly better than that in the placebo group (0%, p<0.0001); the PASI-90 and PASI-100 response rates were 39% and 15%, respectively (89). In the 52-week open-label extension study, efficacy improved with continued treatment, and the PASI-75, PASI-90, and PASI-100 response rates at Week 52 were 78%, 61%, and 39% in the 40 mg BID group, respectively (90).

##### 4.2.1.5 Zasocitinib (TAK-279)

Zasocitinib is a selective TYK2 inhibitor. In a phase IIb randomized, placebo-controlled clinical trial involving patients with moderate-to-severe plaque psoriasis, zasocitinib demonstrated significant dose-dependent efficacy; at Week 12, the PASI-75 response rates were 68% and 67% in the 15 mg and 30 mg QD groups, respectively, which were significantly superior to that in the placebo group (6%, p<0.001) (91). Furthermore, 46% and 33% of patients in the 30 mg group achieved PASI-90 and PASI-100, respectively, with molecular histological further demonstrated suppression of pro-inflammatory gene expressions associated with the IL-23/IL-17 axis (92). Beyond cutaneous improvement, zasocitinib also demonstrated significant benefits in PsA. In another phase IIb study, the ACR20 response rates at Week 12 were 53.3% and 54.2% in the 15 mg and 30 mg QD groups, respectively, which were significantly higher than that in the placebo group (29.2%, p=0.002), suggest that TYK2 inhibition may exert therapeutic effects across both cutaneous and articular inflammatory manifestations in PsA (93).

##### 4.2.1.6 Nomelcitinib (D-2570)

Nomelcitinib is a novel, potent, and oral allosteric TYK2 inhibitor. In a phase II randomized, double-blind, placebo-controlled trial involving patients with moderate-to-severe plaque psoriasis, D-2570 demonstrated high and dose-dependent efficacy. At Week 12, the PASI-75 response rates were 90.0%, 85.4%, and 85.0% in the 18 mg, 27 mg, and 36 mg QD groups, respectively, all of which were significantly superior to the placebo group (12.5%, p<0.001). Furthermore, the PASI-90 response rates were 75.0%, 70.7%, and 77.5%, and the PASI-100 response rates were 40.0%, 39.0%, and 50.0% for the respective D-2570 dose groups, all significantly higher than placebo (5.0% and 2.5%, respectively) (94).

#### 4.2.2 Non-selective JAK inhibitors

##### 4.2.2.1 Tofacitinib

Tofacitinib a JAK1/JAK3 inhibitor with partial activity against JAK2, has been extensively investigated in plaque psoriasis. In the phase III clinical trials (OPT PIVOTAL 1 and 2), the PASI-75 response rate was 39.9% (PIVOTAL 1) and 46% (PIVOTA 2) for tofacitinib 5 mg BID group and 59.2% (PIVOAL 1) and 59.6% (PIVOTAL 2) in the tofacitinib 10 mg BID group (95). In another phase III clinical trial, for patients with moderate-to-severe psoriasis, the PASI-75 response rates were 39.5%, 63.6%, 58.8%, 5.6% in tofacitinib 5 mg BID, tofacitinib 10 mg BID, etanercept 50 mg twice per week (BIW) and placebo, respectively. The results indicated that tofacitinib 10 mg BID was generally comparable to etanercept 50 mg BIW (96). Furthermore, tofacitinib also demonstrated clinical benefits on nail psoriasis (97).

##### 4.2.2.2 Baricitinib

Baricitinib has a strong inhibitory effect on JAK1 and JAK2. In a phase IIb clinical trial, for patients with moderate-to-severe plaque psoriasis, the PASI-75 response rates at Week 12 were 42.9% and 54.1% in the baricitinib 8 mg and 10 mg groups, which were significantly better than placebo (16.1%) (98). Furthermore, a study on a lipid-based topical formulation of baricitinib (BCT-OS) has demonstrated its potential as an alternative treatment for psoriasis in animal models, with potentially higher safety compared to systemic administration (99).

##### 4.2.2.3 Pefecitinib (ASP015K)

Pefecitinib is a pan-JAK inhibitor with relative selectivity for JAK3. In a phase IIa clinical trial, in the treatment of patients with moderate-to-severe plaque psoriasis, the PASI-75 response rates at Week 6 were 31.6%, 26.3%, and 58.8% in the pefecitinib 10 mg BID, 60 mg BID, and 100 mg BID groups, respectively, compared with placebo (3.4%) (100).

#### 4.2.3 Selective JAK1 inhibitors

##### 4.2.3.1 Abrocitinib

In a phase II clinical study, for moderate-to-severe plaque psoriasis, the PASI-75 response rates at 4 weeks were 17%, 17%, 50%, and 60% in the placebo, abrocitinib 200 mg QD, 400 mg QD, and 200 mg BID groups, respectively (101). These dose-dependent efficacy results suggest the possibility that JAK1-mediated upstream cytokine signaling may contribute to psoriasis-associated immune activation.

##### 4.2.3.2 Solcitinib (GSK2586184)

In a phase IIa clinical study, solcitinib demonstrated dose-dependent efficacy in patients with moderate-to-severe plaque psoriasis, with PASI-75 response rates of 13%, 25%, and 57% at Week 12 for solcitinib 100mg BID, 200mg BID, and 400mg BID groups, respectively, which were significantly better than the placebo group (0%, p<0.01) (102).

##### 4.2.3.3 Itacitinib (INCB039110)

In a phase II clinical study, for the treatment of moderate-to-severe plaque psoriasis, itacitinib 200 mg QD and 600 mg QD had a PASI-50 response rate at Week 4 of 66.7% and 81.8%, respectively, which was significantly higher than placebo (8.3%, p<0.05) (103).

### 4.3 Alopecia areata

Alopecia areata (AA) is the most common cause of localized, non-scarring alopecia, caused by an autoimmune attack on hair follicles. Its pathogenesis involves abnormal expression of immune response genes and imbalances in immune cells. The hair follicle is primarily targeted by CD8+ T cells and natural killer (NK) cells. Upregulation of major histocompatibility complex class I (MHC I) molecules and the activation of autoreactive NKG2D CD8+ T cells lead to upregulation of JAK-STAT-dependent inflammatory cytokines, primarily IL-15 and IFN-γ. IFN-γ, in turn, acts through JAK1/2-STAT1 signaling in hair follicle epithelial cells, causing receptor phosphorylation and subsequent IL-15 release, thereby amplifying perifollicular inflammation. Particularly, gamma chain (γc) cytokines such as IL-15, IL-2, and IL-7 bind to JAK1/3 receptors on the surface of NKG2D CD8+ T cells, and promote their activation, survival, and further secretion of IFN-γ, leading to a vicious cycle (104, 105).

The JAK-STAT pathway in the pathogenesis of AA highlights the potential for JAKis as a therapeutic option. Recent evidence suggests that switching to an alternative JAKi represents a viable therapeutic strategy for patients with refractory severe AA, particularly in patients with partial or transient responses to prior JAKi therapy, potentially reflecting differences in JAK selectivity and cytokine pathway modulation (106). The efficacy data of JAKis in AA are detailed below, in which Severity of Alopecia Tool (SALT) score is commonly used for efficacy assessment.

#### 4.3.1 Baricitinib

Baricitinib, the first JAKi approved for the treatment of AA, is currently approved by the FDA and the Chinese National Medical Products Administration (NMPA) (107). In two phase III studies, the percentage of patients achieving a severity of SALT score ≤ 20 after 36 weeks was 16.1%-22.8% in the 2 mg QD baricitinib group and 32.6%-38.8% in the 4 mg QD group, significantly higher than placebo group (3.3%-6.2%, both P < 0.001); by Week 52, this proportion further increased to 36.8%–40.9% (108). Long-term follow-up data demonstrated excellent maintenance of efficacy: among patients who responded at Week 52 and continued treatment, approximately 90% maintained a SALT score ≤ 20 at Week 104 (109); and 89.1% (4 mg group) and 83.6% (2 mg group) maintained this response at week 152 (3 years) (110). Continuous treatment benefited some patients with delayed response; 39.1% of Week 52 mixed responders achieved a SALT score ≤ 20 by Week 104 (109).Continued therapy is crucial for AA. A randomized withdrawal study indicated that among responders who discontinued treatment after 52 weeks, 80% lost treatment benefit by Week 152, compared to only 7% in the continuous treatment group (111). The efficacy of baricitinib in AA may be related to the inhibition of IFN-γ-associated JAK1/2 signaling pathways involved in the collapse of hair follicle immune privilege and perifollicular inflammatory recruitment.

Real-world evidence aligns with clinical trial results. A 48-week study in an Italian cohort reported that 63.2% of patients achieved a SALT score ≤ 20, with significant improvements in quality of life and psychological scores (112). For refractory cases, combination therapies offer new strategies: a retrospective study showed that adding low-dose systemic corticosteroids enabled 60% of patients with inadequate response to baricitinib monotherapy to achieve a SALT score ≤ 20 (113); another study demonstrated that baricitinib combined with oral minoxidil (median dose 2 mg) yielded an overall response rate of 63.2%, with better responses observed in less severe disease subgroups (114).

#### 4.3.2 Ritlecitinib (PF-06651600)

Ritlecitinib, a dual inhibitor of the tyrosine kinase TEC family and JAK3, has been approved by the FDA and NMPA for treating severe AA in patients aged 12 years and older, becoming the first and only oral JAKi approved for adolescent AA patients in China (115). In a phase IIa study, 48 patients with severe AA received ritlecitinib 200 mg QD for 4 weeks, followed by 50 mg QD for 20 weeks, the study showed a SALT-30 response rate of 50% at 24 weeks, significantly better than the placebo group (3%) (116). In the pivotal phase IIb/III ALLEGRO trial, ritlecitinib demonstrated significant efficacy. Results showed that 23% of participants in the 50 mg QD ritlecitinib group achieved a SALT ≤20 at Week 24, compared with only 1.5% in the placebo group (P < 0.001), and this response rate increased to 43.2% through Week 48. Regarding SALT ≤10, 13.4% of participants in the ritlecitinib group achieved this endpoint at Week 24, compared with 1.5% in the placebo group, with the response rate further rising to 31.2% by Week 48. Consistent therapeutic effects were also observed in patients with eyebrow and eyelash involvement (117). *Post hoc* analyses indicated that over 85% of patients who achieved SALT ≤ 20 at Week 24 maintained this response through Week 48 with continued treatment (118). Integrated analysis from the long-term ALLEGRO-LT study revealed that efficacy continued to increase over time; at 24 and 36 months, the proportion of patients achieving SALT ≤ 20 in the continuous 50 mg group significantly improved (119, 120).

Ritlecitinib has also shown promise in special populations and refractory cases. A retrospective review of 18 children under 12 years with severe AA reported that ritlecitinib was well-tolerated, with 66.7% of patients achieving SALT-75 (≥ 75% hair regrowth) (121). Another single-center retrospective study in adults refractory to tofacitinib or baricitinib found that 40.0% achieved SALT ≤ 20 after 24 weeks of switching to ritlecitinib (122). Given the critical role of γc cytokines such as IL-15 in maintaining autoreactive CD8+ T-cell and NK-cell activity, selective inhibition of JAK3-associated signaling may contribute to the therapeutic effects of ritlecitinib in AA.

Real-world evidence from China further supports its clinical utility: a multicenter retrospective analysis (n=100) showed that 34% of patients achieved SALT ≤ 20 at 3 months in clinical practice (123). Another single-center experience reported significant improvements in eyebrow and eyelash assessment scores after 24 weeks of treatment with ritlecitinib 50 mg QD in patients with baseline eyebrow or eyelash involvement (122).

#### 4.3.3 Tofacitinib

Tofacitinib has demonstrated significant efficacy in treating AA across various case reports, case series, and small clinical trials, with doses ranging from 5–15 mg. Hair regrowth typically begins after 6–8 weeks of treatment, and complete hair regrowth was achieved after 3–14 months in responsive patients (124, 125). Recent studies confirmed that an individualized dose-tapering regimen in complete responders effectively maintained disease control, with 95.7% and 93.5% of patients sustaining SALT ≤ 10 at Weeks 24 and 48, respectively; the rate of treatment benefit loss (SALT ≥ 20) remained minimal at 0.0% and 2.2% (126). Furthermore, approximately 25% of patients experienced relapse after a mean of 8.5 weeks following treatment discontinuation, underscoring the necessity for long-term maintenance therapy (125).

#### 4.3.4 Ruxolitinib

In an open-label controlled study, 75 patients with AA were randomized to receive either ruxolitinib 20 mg BID or tofacitinib 5 mg BID for 6 months. The results showed an average SALT score reduction of 95.2 ± 2.69 in the ruxolitinib group and 93.8 ± 3.25 in the tofacitinib group. Hair regrowth was observed significantly earlier in the ruxolitinib group (4.15 ± 3.27 weeks), compared to the tofacitinib group (7.06 ± 2.47 weeks, p = 0.03) (127).

#### 4.3.5 Abrocitinib

Case reports suggest the therapeutic potential of abrocitinib for AA (128–130). In a retrospective study involving 13 patients with AA refractory to prior therapies including systemic corticosteroids, baricitinib, or tofacitinib, oral abrocitinib at doses of 50–200 mg daily led to a significant reduction in SALT score from 74.62 ± 24.96 at baseline to 42.54 ± 35.64, with an overall acceptable safety profile (131).

#### 4.3.6 Upadacitinib

Multiple retrospective studies have demonstrated the significant efficacy of upadacitinib in treating moderate-to-severe and severe AA, including cases refractory to other JAKis, such as baricitinib. A study of 23 patients showed that at 24 weeks, 45.5% of patients achieved partial hair regrowth (≥50% improvement in SALT score) and 36.4% achieved complete regrowth (SALT = 0). By 36 weeks, all continuing patients attained regrowth, with 63.6% achieving complete regrowth (132). In another study of 25 patients, the median SALT score decreased from 50 at baseline to 25 at Week 12 and further to 5 at Week 24, with >50% eyelash and eyebrow regrowth also observed (133). Among 17 patients with AA refractory to prior systemic therapies including other JAKis, upadacitinib treatment for 24 weeks resulted in 88.2% achieving SALT-50, 58.8% achieving SALT-75, and 35.3% achieving SALT-90 (134). Notably, among 9 patients who had previously failed baricitinib, 5 achieved significant hair regrowth with upadacitinib combined with corticosteroid therapy (135). In patients with concomitant AD, efficacy onset was earlier, with the mean SALT score significantly decreasing from 95.1 to 77.6 as early as 4 weeks of treatment (P = 0.0087) (136).

#### 4.3.7 Brepocitinib (PF-06700841)

A phase IIa study confirmed the efficacy of brepocitinib in the treatment of AA, in which 47 patients with severe AA receiving brepocitinib 60 mg daily for 4 weeks, then tapering to a daily dose of 30 mg for another 20 weeks. After 24 weeks of treatment, 64% of patients achieved a SALT 30 response (116). The dual inhibition of JAK1 and TYK2 by brepocitinib may provide broader modulation of inflammatory cytokine signaling involved in AA.

### 4.4 Vitiligo

Vitiligo is a commonly acquired depigmentation disorder characterized by hypopigmented and/or depigmented patches. CD8+ T lymphocytes mediate melanocyte destruction. IFN-γ secreted by CD8+ T cells directly induces melanocyte senescence and apoptosis, promoting surrounding keratinocytes to secrete chemokines such as CXCL9 and CXCL10. These chemokines recruit additional CD8+ T cells to affected skin areas, perpetuating the cycle of melanocyte damage. Both IFN-γ- and IL-15-associated signaling pathway involve JAK-STAT-mediated intracellular transduction, providing a mechanistic rationale for the therapeutic use of JAKis in vitiligo (137, 138). The efficacy data of JAKis in Vitiligo are elaborated below, in which the Vitiligo Area Scoring Index (VASI) and Vitiligo Extent Score (VES) are assessed.

#### 4.4.1 Ruxolitinib

In July 2022, the FDA approved the 1.5% ruxolitinib cream for treating non-segmental vitiligo in patients aged 12 years and older, making it the first JAKi approved for vitiligo in the world (139). In a phase II clinical trial involving adult patients with vitiligo lesions affecting < 20% of body surface area, facial VASI (F-VASI) 50 response rates after 24 weeks were 45%, 50%, 26%, and 32%, in the ruxolitinib 1.5% BID, 1.5% QD, 0.5% QD, and 0.15% QD groups, respectively, compared to the placebo group (3%). After 52 weeks, the F-VASI90 rate was the highest in the ruxolitinib 1.5% BID group, achieving F-VASI50, F-VASI75, and F-VASI90 response rates of 58%, 52%, and 33%, respectively (140). In two phase III clinical studies, F-VASI75 response rates were 29.8% to 30.9% after 24 weeks of treatment with ruxolitinib cream BID in patients with depigmentation affecting ≤10% body surface area, significantly higher than the vehicle control group (7.4% - 11.4%) (141). The long-term extension study (TRuE-V LTE) further evaluated the durability of efficacy. Among patients who achieved F-VASI90 at Week 52 and entered the extension phase, those randomized to continue treatment until Week 104 had an 84.3% probability of maintaining F-VASI75. In contrast, patients randomized to the withdrawal group had a 65.0% probability of maintaining F-VASI75 (142). A meta-analysis of three randomized controlled trials involving 830 patients further confirmed the efficacy of ruxolitinib cream. At 24 weeks of treatment, the improvements in the F-VASI, Total VASI (T-VASI) scores were all significantly superior to those in the placebo group, with the efficacy at 24 weeks being greater than that observed at 12 weeks (143). Regarding refractory areas and combination therapy, a preliminary study of 30 patients with progressive nonsegmental vitiligo demonstrated that 24 weeks of combination treatment with oral upadacitinib (15 mg QD) and topical ruxolitinib cream significantly improved the T-VASI, F-VASI, distal extremities-VASI, and trunk-VASI scores, with 20% and 33% of patients achieving T-VASI50 and F-VASI75, respectively (144). A real-world study in Chinese patients reported a 49.5% F-VASI75 response rate at 24 weeks with ruxolitinib cream, alongside significant improvements in quality of life scores (145).

#### 4.4.2 Ritlecitinib (PF-06651600)

Ritlecitinib is a dual inhibitor of the tyrosine kinase TEC family and JAK3. Studies have shown that ritlecitinib downregulates pro-inflammatory biomarkers in the skin lesions of nonsegmental vitiligo and increases melanocyte products in both the skin and blood of patients (146). In a phase IIb clinical trial involving 364 patients with active nonsegmental vitiligo, various doses of ritlecitinib (200/50 mg, 100/50 mg, 30 mg, or 10 mg) were administered over a 24-week dose-ranging period, followed by 200/50 mg daily in a 24-week extension period. Significant improvements in percent change from the baseline in F-VASI were observed in the ritlecitinib 50 mg, 100/50 mg, and 200/50 mg groups compared with placebo (147, 148). An extension study of this phase 2b trial further evaluated the efficacy of ritlecitinib in combination with narrowband ultraviolet B (NB-UVB) phototherapy. During the 24-week extension period, patients receiving ritlecitinib (200/50 mg) plus NB-UVB achieved a mean percent change from baseline in F-VASI of -57.0% (last observation carried forward), which was greater than the -51.5% (last observation carried forward) observed in the ritlecitinib monotherapy group. The improvement was more pronounced in the observed case analysis (-69.6% vs -55.1% for monotherapy, P = 0.009) (149). Real-world evidence supports these clinical trial findings. A Chinese retrospective study of 11 patients with refractory vitiligo showed that after a mean of 4.18 ± 1.35 months of treatment with oral ritlecitinib (50 mg/day), the VASI score significantly decreased from 4.44 ± 4.16 at baseline to 3.14 ± 2.90, representing a mean improvement of 31.1%. Among them, 9.1% of patients achieved VASI 90, and 18.2% achieved VASI 50 (150). Another retrospective study of 20 patients with vitiligo refractory to either corticosteroids or phototherapy reported that after 24 weeks of ritlecitinib combined with NB-UVB, the mean improvements in T-VASI and F-VASI were 38.3% and 60.0%, respectively. 30% of patients achieved T-VASI 50, and 50% of patients with facial involvement achieved F-VASI 75 (151). The evidence from these studies prompted further investigation of ritlecitinib in Tranquillo phase III clinical program (NCT06163326, NCT06072183, NCT05583526).

#### 4.4.3 Tofacitinib

Oral and topical tofacitinib have shown efficacy in treating vitiligo in various case reports and small-scale studies. In an open-label pilot study, the majority of patients treated with tofacitinib 2% BID for a mean duration of 153 days exhibited significant improvement in facial lesions compared with non-facial lesions (152). A retrospective study of 137 patients with active vitiligo showed that after 3 months of oral tofacitinib 5 mg BID, the median VASI decreased from 3.79 to 2.68, with an overall improvement rate of 29.3%. The Vitiligo Disease Activity score significantly improved from 2.75 ± 1.15 to 1.63 ± 0.66, and 59.1% of patients achieved disease stabilization. Combination therapy with NB-UVB further enhanced efficacy, yielding a significantly higher repigmentation rate at 3 months (38.78%) compared to monotherapy (19.48%) (153). In a 24-week head-to-head randomized controlled trial, oral tofacitinib demonstrated superior durability of response and long-term improvement over oral dexamethasone mini-pulse therapy: while the proportion of patients achieving ≥50% improvement in VES at Week 24 was comparable between groups, the treatment failure rate at Week 12 was significantly lower, and the improvement in VES at Week 36 was significantly greater in the tofacitinib group (154). Topical tofacitinib was also effective. A 16-week intraindividual comparative trial demonstrated that 2% tofacitinib ointment applied BID achieved a higher treatment success rate than 0.1% tacrolimus ointment, with a shorter median time to treatment success and a lower incidence of adverse events (155).

#### 4.4.4 Other JAKi

Other JAKis have also shown potential in treating vitiligo. Several case reports indicate the efficacy of oral baricitinib and topical digolitinib for vitiligo, particularly in patients with refractory disease previously treated with TCS or NB-UVB phototherapy (156). Other JAKis currently under investigated for vitiligo include delgocitinib, cerdulatinib (SYK/JAK dual-target inhibitor) gel, and ifidancitinib (JAK1/JAK3 inhibitor) (157).

### 4.5 Overall safety of JAK inhibitors

The most frequent adverse events associated with oral JAKis include upper respiratory infections, nasopharyngitis, nausea, headache, acne and laboratory abnormalities such as lipid elevation and transient changes in blood cell counts. However, more severe adverse events have been reported, including major adverse cardiovascular events, malignancy, venous thromboembolism, and all-cause mortality. Therefore, physicians should carefully evaluate individual patient risk factors before prescribing system JAKis and inform patients about the FDA’s black box warning (158, 159). Importantly, the current FDA boxed warning for JAKis was primarily derived from safety data in rheumatoid arthritis populations with relatively high baseline cardiovascular risk, and whether these risks are directly generalizable to dermatologic populations remains under ongoing investigation. The safety profiles of different JAKis may vary according to JAK selectivity, route of administration, treatment duration, and underlying disease characteristics.

While clinical trials are reliable for assessing efficacy, they are less dependable for identifying adverse events because of limited sample sizes and follow-up durations. Conversely, meta-analyses and real-world studies may better reflect class-wide safety signals, although differences between individual JAKis should still be interpreted cautiously. A meta-analysis of dermatology-specific phase III clinical trials reported venous thromboembolism rates ranging from none to 0.1–0.5%, compared to no events in the placebo groups (160). The rates of cardiovascular events ranged from none to 0.4–1.2%, compared to no events to 0.5–1.2% in placebo groups, indicating there is no strong signal for cardiovascular events. Overall, continued vigilance is needed to fully understand the safety profile of JAKis and to ensure informed clinical decision-making.

## 5 The concept and future perspectives of “JAK-STAT pathway-associated skin diseases

Over the past decade, JAKis have emerged as important therapeutic options for a broad spectrum of inflammatory skin diseases. These include not only approved indications (**Table 4**), such as AD, psoriasis, AA, and vitiligo, but also off-label applications and ongoing clinical studies investigating additional dermatologic indications. These advances have benefited from an in-depth understanding of disease immunopathogenesis, particularly the central role of JAK-STAT-dependent cytokine signaling in immune activation, tissue inflammation, and disease chronicity.

**Table 4:** Characteristics of different JAKi currently approved for dermatological use.

JAKi	Targets	Approved indications for skin diseases worldwide
Tofacitinib	JAK1/3>JAK2	PsA
Baricitinib	JAK1/2	AD, AA
Upadacitinib	JAK1>JAK2>JAK3	AD, PsA
Abrocitinib	JAK1	AD
Ruxolitinib (topical)	JAK1/2	AD, Vitiligo
Delgocitinib (topical)	JAK1/2/3, TYK2	AD, CHE
Deucravacitinib (BMS-986165)	TYK2	Plaque Psoriasis
Ivarmacitinib (SHR0302) (oral)	JAK1	AD

PsA, Psoriasis/psoriatic arthritis; AD, atopic dermatitis; AA, alopecia areata; CHE, chronic hand eczema.

For inflammatory skin diseases with marked immunologic complexity and heterogeneity, therapeutic strategies targeting a single cytokine are often insufficient to comprehensively suppress disease-associated immune networks. JAKis can meet this need by acting downstream of the cytokine signaling pathway. Additionally, in cases of comorbid inflammatory diseases, such as AD co-occurring with AA or rheumatoid arthritis (RA), where overlapping innate and adaptive immune pathways are simultaneously involved. In these conditions, the availability of JAKis that can target multiple cytokines may offer a synergistic therapeutic effect on comorbidities. Despite the potential for cross-talk among different diseases within the JAK-dependent cytokine module, therapeutic responses to JAKis may be similarly overlapping, even when considering the diverse Th immune subsets involved.

We propose the concept of “JAK-STAT pathway-associated skin diseases”, encompassing a group of diseases in which JAK-STAT pathway-related cytokines play a pivotal role in various stages of the disease, including initiation, progression, recurrence, and comorbidities. This concept may provide a mechanistic framework for understanding shared immunopathologic features among inflammatory skin diseases and may further support a pathway-oriented therapeutic strategy in clinical practice, and subsequently determining whether patients might benefit from JAKi treatment based on current evidence.

**Table 5** categorizes the high correlation of the JAK-STAT pathway with common skin diseases, which may provide a useful reference for individualized therapeutic decision-making. After deciding to use a JAKi regimen, the next step is to select the most appropriate JAKi according to disease characteristics, cytokine profiles, comorbidities, route of administration, and current evidence. Options include pan-JAKis, or selective JAKis, or a dual inhibitor of JAK with other kinases. For example, the selective TYK2 inhibitors may meet the therapeutic need of plaque psoriasis, but for patients with PsA, upadacitinib or tofacitinib may be more advantageous, due to involvement of a broader range of cytokines.

**Table 5:** Level of evidence of “JAK-STAT pathway-associated skin disease”.

Strength of correlation with JAK-STAT pathway	Level of evidence	Skin diseases
Grade A	Meta-analysis, systemic review	Atopic Dermatitis, Vitiligo, Chronic hand eczema
Grade B	Phase III clinical trial	Psoriasis/psoriatic arthritis, Alopecia areata
Grade C	Case series, real-world study	Dermatomyositis, Sarcoidosis, Chronic urticaria, Systemic Lupus erythematosus, Lichen planus
Grade D	Expert experience, Case report	Allergic contact dermatitis, Autoimmune bullous diseases, Urticarial vasculitis, Livedoid vasculopathy

## 6 Limitations and future perspectives

Despite the potential of the JAK-STAT pathway framework proposed in this review for clinical application in dermatology, several limitations must be acknowledged, and future clinical practice and research need to further address the following issues. Firstly, the extant evidence is largely based on short- to medium-term clinical trials, and long-term safety and efficacy maintenance data for the majority of JAKis in dermatology patients remains insufficient. Secondly, the paucity of direct comparative studies between different JAKis hinders precise evaluation of the relative merits of varying target selectivities (e.g., JAK1, TYK2, and JAK1/2 inhibitors) in specific diseases or phenotypes. Thirdly, although an attempt has been made to explain treatment responses from the perspective of “JAK-dependent cytokine modules,” these mechanistic inferences are largely based on indirect clinical observations. At present, there is an absence of clinically validated biomarkers to guide patient stratification and precision treatment selection. Fourthly, there are certain limitations that persist at the clinical application level, and several practical issues that remain to be clarified. These include the following: optimal dosing regimens (dose, duration) for different diseases and disease stages; safe and effective drug tapering strategies; management of relapse following discontinuation; and follow-up treatment plans for patients with poor initial treatment response. Furthermore, a more nuanced contextual interpretation of the FDA’s “black box warning” is still required. Finally, existing clinical data are primarily concentrated in the adult population. In infants and young children, who bear an equally heavy disease burden, the efficacy, safety, and age-specific immunological effects of JAKis have not yet been fully validated. The lack of substantial data in this pediatric population remains a significant gap, limiting the extensive utilization of JAKi in this demographic. Addressing these challenges through longitudinal cohort studies and carefully designed clinical trials will be pivotal to transitioning from empirical treatment to personalized precision medicine in dermatology.

In summary, while JAKis have expanded the treatment landscape for inflammatory skin diseases, it remains crucial to continue developing highly selective inhibitors, identifying predictive biomarkers, and evaluating long-term safety. The framework of “JAK-STAT pathway-related skin diseases” is expected to drive the development of more mechanism-driven, personalized treatment models in the future and may extend their application to a broader patient population.
