# Supplementary material for: JAK-STAT pathway-associated skin diseases: a refined functional framework for inflammatory skin diseases
Source: Front Immunol. 2026 Jun 18;17:1851255. doi: 10.3389/fimmu.2026.1851255 (PMC13322818; doi:10.3389/fimmu.2026.1851255)
Supplement: Supplementary file 1 [file Table1.pdf]

Table S1. Other dermatological uses of JAK inhibitors

**Skin disease**	**Drug**	**Evidence level**
Systemic sclerosis	Tofacitinib<br>Baricitinib	Systematic review,<br>real-world study
Localized scleroderma	Tofacitinib<br>Delgocitinib<br>Upadacitinib	Systematic review,<br>case report
Hidradenitis suppurativa	INCB054707<br>Upadacitinib<br>Brepocitinib<br>Zimlovisertib<br>Ropsacitinib	Phase II studies,<br>real-world study
Necrobiosis lipoidica	Ruxolitinib (topical)<br>Upadacitinib	Case report
Rosacea	Tofacitinib<br>Upadacitinib<br>Abrocitinib<br>Ruxolitinib (topical)	Case series,<br>case report
Cutaneous amyloidosis	Tofacitinib	Case report
Granuloma annulare	Tofacitinib<br>Upadacitinib	Case series,<br>case report

	Abrocitinib<br>Deucravaitinib<br>Ruxolitinib (topical)	
Behcet's disease	Tofacitinib<br>Filgotinib<br>Upadacitinib	Phase II studies,<br>case report
Chronic actinic dermatitis	Tofacitinib<br>Upadacitinib<br>Baricitinib	Case series
Epidermolysis bullosa pruriginosa	Baricitinib<br>Upadacitinib<br>Tofacitinib<br>Ritlecitinib	Case series,<br>case report
Darrier's disease	Baricitinib<br>Tofacitinib	Case report
Hailey-Hailey disease	Abrocitinib<br>Upadacitinib<br>Ruxolitinib (topical)	Case series,<br>case report
Keloids	Ruxolitinib (topical)<br>Tofacitinib<br>Upadacitinib	Open-Label, uncontrolled, single-Arm Clinical Trial,<br>case report

Palmoplantar pustulosis	Tofacitinib<br>Upadacitinib<br>Ruxolitinib	Retrospective study,<br>case series
Polyarteritis nodosa	Tofacitinib<br>Baricitinib	Case report
Chronic mucocutaneous candida infections	Baricitinib	Case series
Prurigo nodularis	Tofacitinib<br>Baricitinib<br>Upadacitinib<br>Abrocitinib	Case series,<br>case report
Netherton Syndrome	Abrocitinib<br>Upadacitinib<br>Tofacitinib	Case report
